# Supplementary material for: Anti-Proteolytic Peptide R7I Protects the Intestinal Barrier and Alleviates Fatty Acid Malabsorption in Salmonella typhimurium-Infected Mice
Source: Int J Mol Sci. 2023 Nov 16;24(22):16409. doi: 10.3390/ijms242216409 (PMC10670956; doi:10.3390/ijms242216409)
Supplement: Supplementary file 1 [file ijms-24-16409-s001.zip › ijms-2681093-supplementary.pdf]

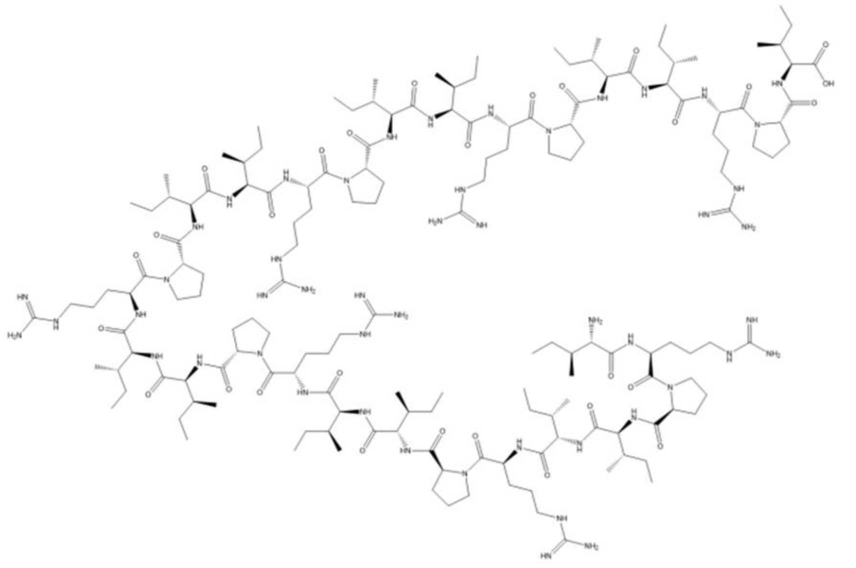

Figure S1: Structure of R7I

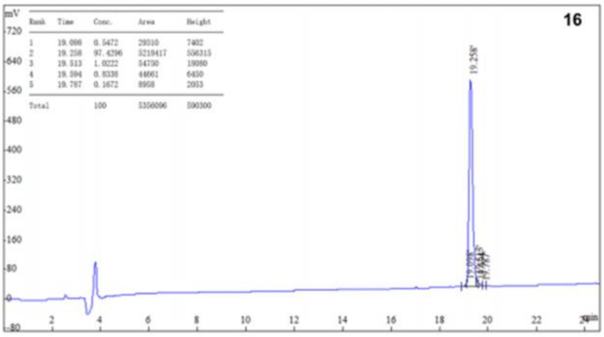

Rank	Time	Conc.	Area	Height
1	19.098	0.5472	29310	7402
2	19.258	97.4296	5218417	556315
3	19.513	1.0222	54750	18080
4	19.594	0.8338	44661	6450
5	19.787	0.1672	8958	2053
Total		100	5356096	590300

Figure S2:HPLC spectra of R7I. HPLC with a column of SHIMADZU Inertsil ODS-SP 4.6×250 mm×5 µm, 214 nm, 10 µL column using a nonlinear water/acetonitrile gradient containing 0.1% Trifluoroacetic at a flow rate of 1.0mL/min

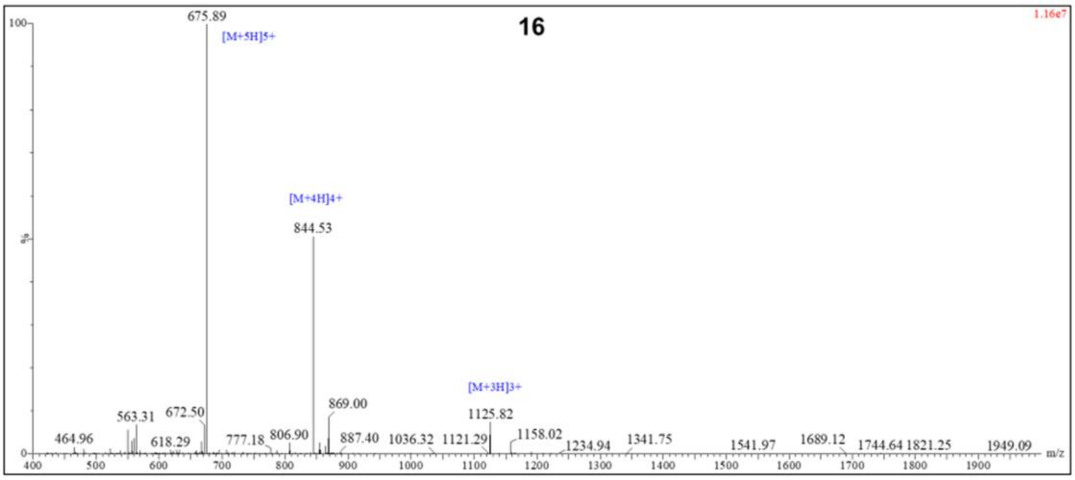

Figure S3: MALDI-TOF MS of R7I
